# Takotsubo Syndrome Associated with Neurally Mediated Reflex Syncope: A Meta-summary of Case Reports and Literature Review

**DOI:** 10.31083/j.rcm2507264

**Published:** 2024-07-15

**Authors:** Vincenzo Russo, Filippo Pecori, Nicola Colalillo, Nicola Massimo, Giovanni Battista Valentino, Angelo Comune, Erika Parente, Gerardo Nigro

**Affiliations:** ^1^Department of Translational Medical Sciences, Cardiology and Syncope Unit, University of Campania “Luigi Vanvitelli” – Monaldi Hospital, 80131 Naples, Italy

**Keywords:** Takotsubo syndrome, neurally mediated reflex syncope, head up tilt test, implantable loop recorder, emotional stress, vasovagal syncope

## Abstract

**Background:**

Neurally mediated reflex syncope (NMRS) has been recently described as a possible trigger of Takotsubo syndrome (TTS). There are few data in the literature about this association.

**Methods:**

In the present meta-summary, 6 case reports describing patients who experienced TTS following an NMRS episode were included. Patient characteristics, triggers and type of syncope were collected.

**Results:**

A total of 7 patients with a median age of 63.4 years (interquartile range, IQR: 47.5–76) were evaluated; 71.4% were females, mainly in the menopausal state (80%). The TTS triggers were: vasovagal syncope in 6 patients (85.7%) and situational syncope in 1 patient (14.3%). 2 patients underwent a comprehensive clinical evaluation which showed a cardioinhibitory response.

**Conclusions:**

NMRS due to sudden orthostatism and emotional stress, mainly with a cardioinhibitory response, has been associated with the onset of TTS, in particular among female patients in a menopausal state.

## 1. Introduction

Takotsubo syndrome (TTS) is a condition of transient left ventricular dysfunction with typical regional wall motion abnormalities that leads to acute heart failure in the absence of significant culprit epicardial coronary artery disease [1]. Symptoms resembling an acute myocardial infarction, such as chest pain and/or shortness of breath; electrocardiographic irregularities (ST-segment elevation or depression and/or T-wave inversion); and increased serum cardiac troponin levels are considered the most common clinical features of the disease presentation.

TTS is frequently triggered by emotional or physical stress [2]. Recently some cases of neurally mediate reflex syncope (NMRS) triggering TTS have been described [3, 4, 5, 6, 7, 8]. Our meta-summary of case reports aimed to characterize patients who experienced TTS following a NMRS episode.

## 2. Methods

**Fig. 1.**
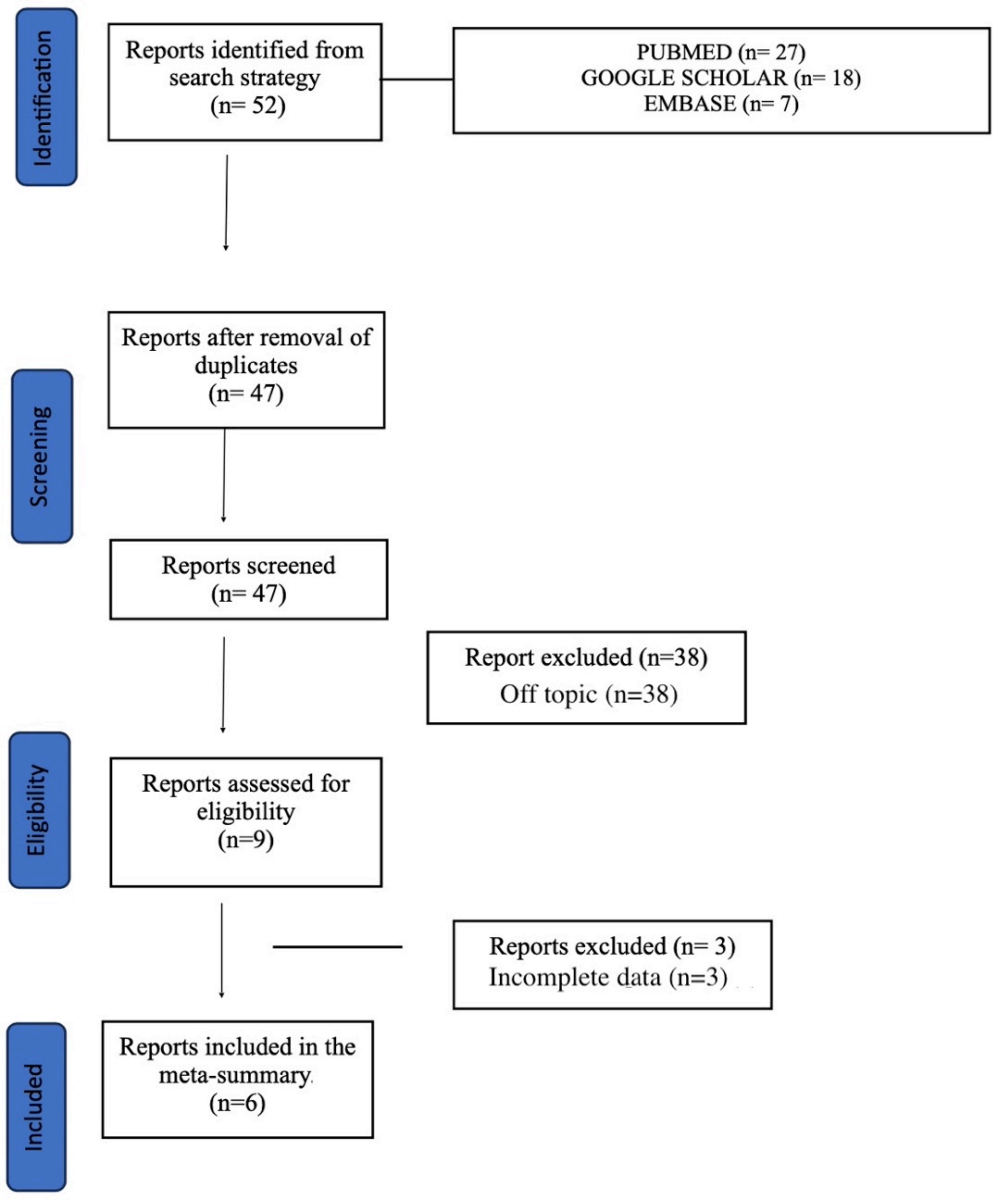
**Flow diagram of the study selection**.

We searched for case reports published in Pubmed, Google Scholar and EMBASE from 2008 to 2023 using the following keywords: “Takotsubo Syndrome”, “Vasovagal syncope”, “Reflex neurally-mediated syncope”. We considered English original reports only; double papers were ruled out. All titles and abstracts were independently screened by different researchers (GBV, FP, NC, NM). Any discrepancies were resolved by discussion and consensus (VR, EP, AC). Finally, 6 eligible case reports/case series were included in our meta-summary. The diagram of study selection is shown in Fig. 1.

## 3. Results

A total of 7 patients with diagnosed TTS triggered by NMRS were examined. The median age was 65 (interquartile range, IQR: 47.5–76) years and 71.4% were female. 4 out of 5 women were in the menopausal state. The triggers of TTS were: vasovagal syncope in 6 patients (85.7%) and situational syncope in 1 patient (14.3%). Two patients underwent a comprehensive clinical evaluation, including a head up tilt test (HUTT) and implantable loop recorder (ILR), which showed a cardioinhibitory response. The TTS was characterized by reduced left ventricular ejection fraction in 3 patients (42.8%) and hypokinesis of the mid-left ventricle without reduction of ejection fraction in 1 case (14.3%). No sudden cardiac death or life-threatening arrhythmias were reported. The median time of hospitalization was 53.3 (IQR: 12–90) days. Table 1 (Ref. [3, 4, 5, 6, 7, 8]) shows the clinical features of the study population.

**Table 1.** **Clinical features of the study population**.

Year	Authors	Age (years)	Gender	Menopause	Comorbidities	Type of syncope	Trigger	Complications	Hospitalization (days)
2011	Mohammad M. *et al*. [3]	60	Female	Yes	Hypertension, dyslipidemia	Vasovagal	Abdominal pain	N/A	N/A
2009	Lee L. *et al*. [4]	81	Female	Yes	COPD, depression	Vasovagal	Sudden orthostatism	Reduced LVEF	14
2018	Setwala A. *et al*. [5]	80	Male	-	N/A	Vasovagal (CI response at ILR)	N/A	N/A	30
2018	Setwala A. *et al*. [5]	35	Female	No	N/A	Vasovagal	Orthostatism	Hypokinesis of mid LV	30
2020	Ashfaq A. *et al*. [6]	58	Male	-	COPD, hypertension	Situational	Gastrointestinal stimulation	N/A	60
2020	Orphanou N. *et al*. [7]	70	Female	Yes	Hypertension	Vasovagal	Happiness	Reduced LVEF	6
2022	Raccis M. *et al*. [8]	71	Female	Yes	Hypertension, dyslipidemia	Vasovagal (CI response at HUTT)	N/A	Reduced LVEF	180

Abbreviations: COPD, chronic obstructive pulmonary disease; LVEF, left ventricular ejection fraction; CI, cardioinhibitory; HUTT, head up tilt test; ILR, implantable loop recorder; N/A, not available; LV, left ventricular.

## 4. Discussion

Although the aetiology of TTS is not entirely known, a central role of the autonomic nervous system has been hypothesized [9]. In particular, the activation of the sympathetic nervous system and the excessive release of catecholamines seem to play a crucial role in the TTS pathogenesis leading to multivessel epicardial spasm, microvasculature dysfunction and cardiotoxicity [1]. Several experimental studies investigated the neurohormonal changes during HUTT and its role in vasovagal syncope. According to their results, epinephrine (Epi) levels increased early during the standing posture to a greater extent in fainters than controls; moreover, before syncope, the Epi level continued to heighten, reaching values up to 15 times higher than baseline [10, 11, 12, 13]. Furthermore, a greater increase of Epi levels from baseline to 2 min of HUTT has been associated with a shorter time to syncope [14, 15]. According to our results, vasovagal syncope due to sudden orthostatism and emotional stress, mainly with a cardioinhibitory response, seemed to be a possible trigger of TTS; female patients in the menopausal state showed this association more frequently. The marked increase in circulating epinephrine associated with vasovagal syncope may represent the pathophysiological link between NRMS and TTS. The high prevalence of vasovagal syncope among the general population and the low number of cases in which it seems to be the onset of TTS suggests the rarity of this association, mainly described in menopausal women.

## 5. Limitations

The screening of only English language papers and the few numbers of included cases certainly pose a constraint; however, the present article is the first meta-summary exploring the association between TTS and NMRS. It is difficult to define the causal relationship between TTS and NMRS, since they share the same triggers (i.e., emotional stress is the trigger in 27.7% of TTS), moreover, the syncope due to sudden and transient left ventricular dysfunction may be the first manifestation of TTS in 8% of cases [2].

## 6. Conclusions

NMRS due to sudden orthostatism and emotional stress, mainly with a cardioinhibitory response, seems to be a possible trigger of TTS, in particular among female patients in the menopausal state.

## Data Availability

The data that support the findings of this study are available from the corresponding author, upon reasonable request.
